# Concomitant autoimmunity may be a predictor of more severe stages of endometriosis

**DOI:** 10.1038/s41598-021-94877-z

**Published:** 2021-07-28

**Authors:** Valeria Stella Vanni, Roberta Villanacci, Noemi Salmeri, Enrico Papaleo, Diana Delprato, Jessica Ottolina, Patrizia Rovere-Querini, Stefano Ferrari, Paola Viganò, Massimo Candiani

**Affiliations:** 1 Gynecology/Obstetrics Unit, IRCCS San Raffaele Scientific Institute, 20132 Milan, Italy; 2 Vita-Salute San Raffaele University, 20132 Milan, Italy; 3 Division of Immunology, Transplantation & Infectious Diseases, IRCCS San Raffaele Scientific Institute, 20132 Milan, Italy; 4 Reproductive Sciences Lab, Obstetrics and Gynecology Unit, IRCCS San Raffaele Scientific Institute, 20132 Milan, Italy

**Keywords:** Adaptive immunity, Autoimmunity, Innate immunity

## Abstract

Pathogenesis of endometriosis is still unclear and a role of both innate and adaptive immune system has been postulated. Some recent findings have revealed an increased risk to have concomitant autoimmune disease in women with endometriosis, but no study so far has investigated whether this association could affect endometriosis severity and stage. We retrospectively reviewed medical patients’ notes of women with a confirmed diagnosis of endometriosis who referred to our endometriosis outpatient clinic between January 2015 and December 2019. Cases (endometriosis and an autoimmune disease) were matched in a 1:3 ratio by age and study period with controls (endometriosis without history of autoimmunity). At univariate logistic analysis, concomitant autoimmunity (OR 2.63, 95% CI 1.64–4.21, p < 0.001) and the number of laparoscopic procedures performed (OR 2.81, 95% CI 1.45–5.43, p = 0**.**002) emerged as factors significantly associated with the likelihood of stage IV endometriosis. In the multivariate logistic regression model, concomitant autoimmunity remained a significant predictor of stage IV endometriosis (OR 2.54, 95% CI 1.57–4.10, p = 0.004), whereas the association between the number of laparoscopic procedures performed and stage IV endometriosis was found to be of borderline-significance (OR 2.70, 95% 1.37–5.30, p = 0.050). Our findings suggest that endometriosis is more severe in patients who are also affected by autoimmune disturbances after controlling for relevant confounders.

## Introduction

Endometriosis, traditionally defined as the presence of endometrial-like tissue outside the uterine cavity, is a chronic gynaecological disease which affects 2–22% of reproductive age women^1^. It can be asymptomatic or associated with infertility, chronic pelvic pain and dyspareunia^2^. Although its pathogenesis has not been completely clarified, endometriosis is known to be a hormone dependent chronic inflammatory disease^3^ characterized by activation of both innate and adaptive immune system^4,5^. Indeed, researches made during the last two decades have found plenty of immunological abnormalities. An increased production of pro-inflammatory cytokines/chemokines, a higher concentration of peritoneal macrophages^4^, alterations in B cell activation, and immunological abnormalities in T/B cell function^6^ are only few examples of this immunological dysfunction. Also, some genes involved in the immune response were found to be differently expressed in peripheral leukocytes of women with endometriosis (stage III-IV) similarly to other non-gynaecologic and chronic inflammatory conditions^7^.

A recent systematic review and meta-analysis by Shigesi and coworkers^8^ has found an increased risk of comorbidity of autoimmune diseases including systemic lupus erythematosus (SLE), Sjögren’s syndrome (SS), rheumatoid arthritis (RA), autoimmune thyroid disorders (ATD), celiac disease (CLD), multiple sclerosis (MS), inflammatory bowel disease (IBD), and Addison’s disease in women with endometriosis, despite a high risk of bias related to the low quality of included studies exists and was reported by the authors. Indeed, endometriosis seems to share features characteristic of autoimmune diseases such as an increased presence of auto-antibodies^9^ Serum of endometriosis patients contains a high level of anti-macrophage colony stimulating factor antibodies (anti-GM-CSF Ab) which correlates with severity and number of lesions^10^. GM-CSF, which is a hematopoietic growth factor produced by many different cells including endometrial cells, plays a crucial role in linking innate and adaptive immunity^10,11^.

Some of genetic polymorphisms in the autoimmunity genes have also been investigated in endometriosis, although with inconsistent results^12^. These findings have led to the idea of endometriosis as a unique immunological scenario outlining the plausible association between endometriosis and immunity/autoimmunity^6^. Nonetheless, no study has so far investigated whether the observed association between endometriosis and autoimmunity might be related to the severity of endometriosis.

To shed more light into the possible link between endometriosis and autoimmunity, we designed a matched case–control study to investigate whether the concomitant presence of autoimmune diseases is associated with different stages of endometriosis.

## Results

Baseline characteristics of the whole cohort of women included is shown in Table 1.

**Table 1:** Baseline characteristics of patients (*n* = 384).

Baseline characteristics	
Age at evaluation (years)	38.8 ± 6.1
Age at diagnostic LS (years)	30.9 ± 5.6
Age at symptoms onset (years)	17 [13–25]
Time from LS (years)	7.9 ± 5.7
Number of surgical procedures	1 [1–1]
Number of clinical evaluations	2 [1–2]
**r-AFS stage**	
I	12 (3.1%)
II	37 (9.7%)
III	182 (47.5%)
IV	152 (39.7%)
**Type of endometriosis**	
OMA	268 (69.9%)
SPE	220 (57.4%)
DIE	140 (36.6%)
**Hormone therapy**	
Any	180 (47%)
None	124 (32.2%)
Not known	80 (20.8%)

Values are mean ± SD or median [25%-75%] or n (%).

LS, laparoscopic surgery; r-AFS, revised American Fertility Society; OMA, ovarian endometrioma; SPE, superficial peritoneal endometriosis; DIE, deep infiltrating endometriosis.

Comparison between women with endometriosis and concomitant autoimmune disease (cases, n = 96) and women with endometriosis and negative autoimmune status (controls, n = 268) is shown in Table 2, where also the specific autoimmune diseases found in our population are shown.

**Table 2:** Baseline characteristics of patients according with the autoimmunity status (n_1_ = 96; n_2_ = 288).

Baseline characteristics	Cases (*n*_1_ = 96)	Controls (*n*_2_ = 288)	*p*-value
Age at evaluation (years)	38.7 ± 6.1	38.7 ± 6.1	0.987
Age at diagnostic LS (years)	31.1 ± 6.2	30.1 ± 5.4	0.761
Age at symptoms onset (years)	16 [14–20]	18 [13–25]	0.960
Time from LS (years)	7.7 ± 5.9	8.0 ± 5.7	0.703
Number of surgical procedures	1 [1–1]	1 [1–1]	0.166
Number of clinical evaluations	2 [2–3]	2 [1–2]	**0.0001**
**r-AFS stage**			
I	2 (2.1%)	10 (3.5%)	0.495
II	7 (7.3%)	30 (10.5%)	0.364
III	32 (33.3%)	150 (52.3%)	**0.001**
IV	55 (57.3%)	97 (25.3%)	**0.000**
**Type of endometriosis**			
OMA	75 (78.1%)	193 (67.2%)	0.096
SPE	45 (46.9%)	175 (61%)	**0.016**
DIE	43 (44.8%)	97 (33.8%)	0.053
**Hormone therapy**			
Any	43 (44.8%)	137 (47.7%)	0.617
None	32 (33.3%)	92 (32.1%)	0.817
Not known	21 (21.9%)	58 (20.2%)	0.727
**Concomitant autoimmune disease**			**0.000**
None		287 (100%)	
ATD	57 (59.4%)		
T1D	10 (10.4%)		
IBD	9 (9.4%)		
CLD	1 (1%)		
LES/APS	5 (5.2%)		
RA	6 (6.3%)		
Fibromyalgia	2 (2.1%)		
SS	1 (1%)		
MS	4 (4.2%)		
Psoriasis	1 (1%)		
Multiple	15 (15.6%)		

Values are mean ± SD or median [IQR] or n (%).

Bold values denote statistical significance at the *p* < 0.05 level.

LS, laparoscopic surgery; r-AFS, revised American Fertility Society; OMA, ovarian endometrioma; SPE, superficial peritoneal endometriosis; DIE, deep infiltrating endometriosis; ATD, autoimmune thyroid disorders; T1D, type 1 diabetes; IBD, inflammatory bowel diseases; CLD, coeliac disease; SLE, systemic lupus erythematosus; APS, antiphospholipid syndrome, RA, rheumatoid arthritis; SS, systemic sclerosis; MS, multiple sclerosis.

**Figure 1:**
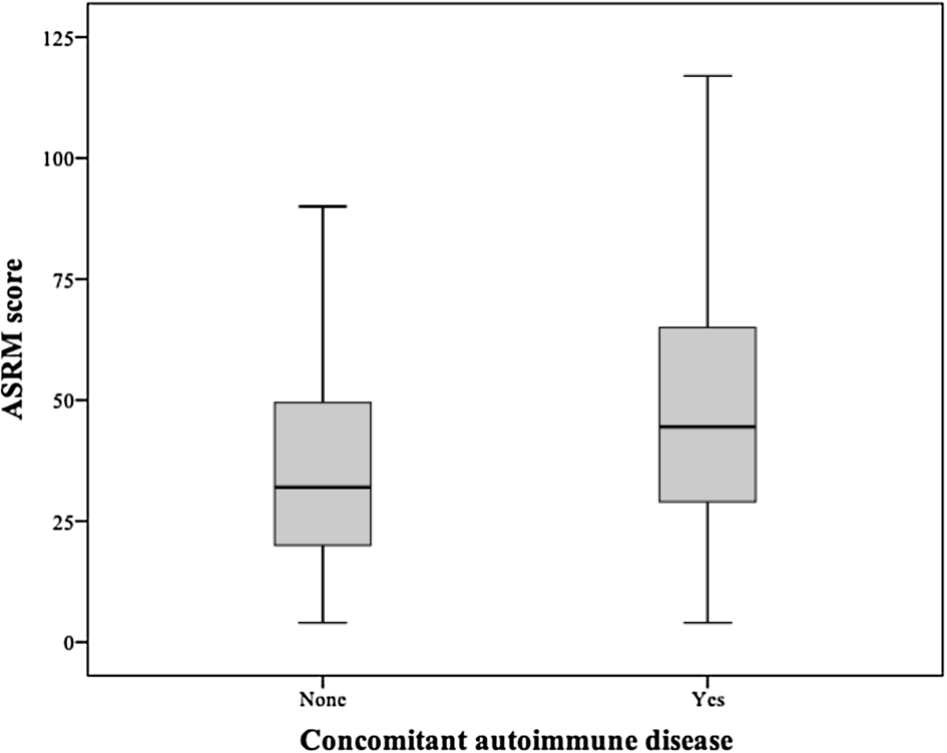
Boxplots of ASRM score according with autoimmunity status. We compared mean ASRM score between women without autoimmunity and women with concomitant autoimmune disease. ASRM score, American Society for Reproductive Medicine.

The two groups were comparable in terms of age at laparoscopy (31.1 ± 6.2 versus 30.1 ± 5.4 in cases and controls respectively, p = 0.761) and time interval between laparoscopy and inclusion in the study (7.7 ± 5.9 versus 8.0 ± 5.7 years in cases and controls respectively, p = 0.703).The proportion of women using hormonal treatment for endometriosis at the time of inclusion in the study was also comparable between the two groups (44.8% versus 47.7% in cases and controls respectively, p = 0.617). Compared to women without autoimmunity, women with concomitant autoimmune disease had a higher mean ASRM score (51 versus 39, p = 0.001, Fig. 1) and presented more often with stage IV endometriosis (25.3% versus 57.3%, p < 0.001) and less frequently with stage III disease (33.3% vs 52.3%, p = 0.001).

At univariate logistic analysis, concomitant autoimmunity (OR 2.63, 95% CI 1.64–4.21, p < 0.001) and the number of laparoscopic procedures performed (OR 2.81, 95% CI 1.45–5.43, p 0**.**002) emerged as factors significantly associated with the likelihood of stage IV endometriosis (Table 3).

**Table 3:** Logistic regression analysis of independent risk factors for stage IV of endometriosis disease.

Parameters	Univariate logistic regression			Multiple logistic regression		
	Odds ratio	95% CI^a^	*p*-value	Odds ratio	95% CI^a^	*p*-value
Age at evaluation (years)	1.01	0.97–1.04	0.65			
Age at diagnostic LS (years)	0.97	0.94–1.01	0.23			
Age at symptoms onset (years)	0.97	0.94–1.01	0.19			
Time from LS (years)	1.01	0.97–1.05	0.48			
Number of LS procedures*	2.81	1.45–5.43	**0.002**	2.70	1.37–5.30	**0.050**
Number of clinical evaluations	0.91	0.79–1.05	0.20			
Autoimmunity*	2.63	1.64–4.21	**0.000**	2.54	1.57–4.10	**0.004**
Hormone therapy	1.26	0.95–1.67	0.11			

LS, laparoscopic surgery.

*Variables included in the multiple logistic regression analysis.

Bold values denote statistical significance at the *p* ≤ 0.05 level.

^a^95% confidence intervals.

In the multivariate logistic regression model, concomitant autoimmunity remained a significant predictor of stage IV endometriosis (OR 2.54, 95% CI 1.57–4.10, p = 0.004), whereas the association between the number of laparoscopic procedures performed and the diagnosis of stage IV endometriosis was found to be borderline-significant (OR 2.70, 95% 1.37–5.30, p = 0.050, Table 3).

## Discussion

To the best of our knowledge, our study has been the first that investigated whether the presence of concomitant autoimmunity is associated with advanced stage of disease in women with endometriosis, and suggests that endometriosis is more aggressive in patients who are also affected by autoimmune disturbances after controlling for relevant confounders. The coexistence of endometriosis and autoimmunity is a well-known occurrence. Based on previous studies and meta-analyses, women with endometriosis are at higher risk for autoimmune diseases including SLE, SS, RA, CD, MS, or IBD compared to healthy controls^8^. Nonetheless, none of the previous studies have addressed whether the coexistence of the two conditions is associated with a more severe endometriosis. Our results are thus the first to suggest that comorbid autoimmune conditions in patients with endometriosis might be considered as a risk factor for stage IV disease.

While our novel results might outline an interplay between endometriosis progression and autoimmunity, causal relationships cannot be inferred and conclusions about whether more severe endometriosis is a consequence of concomitant autoimmunity cannot be drawn. Common pathological mechanisms such as macrophage dysfunction and impaired clearance of apoptotic cells^13^ might underlie the two conditions, or systemic and local tissue damage resulting from autoimmune diseases might have untoward effects on endometriosis progression. Nonetheless, our results might have several clinical implications: on one hand, closer surveillance or different treatment strategies for endometriosis might be needed in patients with concomitant autoimmunity. The diagnosis of a concomitant autoimmune disease might for example indicate that the patient is at higher risk for fibrotic progression—a common and highly pathogenic feature of both stage IV endometriosis^14,15^ and autoimmune diseases. Fibrosis prevention is a putative target for early immune-modulatory therapies^16^. Thus, the identification of concomitant autoimmune disease as an early risk factor for fibrotic progression in patients with endometriosis would be of outmost clinical importance. On the other hand, if our results were confirmed by larger studies, screening for autoimmunity might become indicated in patients with endometriosis and targeted studies focused on immune-modulatory therapies in this subgroup of patients might become valuable.

Our study also presents some limitations. First, its retrospective nature. While only women with a both surgical and histological diagnosis of endometriosis were included, medical patients’ notes regarding concomitant autoimmune diseases severity and stage were less detailed and for i.e. did not comprise information about age of onset. In addition, our study has a moderate sample size in this setting (100–1000 patients^8^) and confirmation of our novel results in investigations with a large sample size is needed.

## Conclusions

Our findings suggest that the presence of concomitant autoimmune disease in patients with endometriosis might be considered as a risk factor for stage IV disease severity.

## Methods

This was a retrospective case–control study carried out at the department of Obstetrics and Gynecology, IRCCS San Raffaele Scientific Institute, Milan. We reviewed medical notes of all women of reproductive age and surgically/histopathologically confirmed diagnosis of endometriosis who referred to our endometriosis clinic from January 2015 to February 2019. Among them, we selected women with concomitant autoimmunity (cases), whose presence was assessed by medical interview at the time of visit. Whenever a patient self-reported a diagnosis of autoimmune disease, the presence of such condition was further investigated by retrieving previous blood tests for auto-antibodies or previous rheumatological records. We considered any of the following autoimmune diseases: autoimmune thyroiditis, celiac disease (CD), systemic lupus erythematous (SLE), inflammatory bowel disease (IBD), multiple sclerosis (MS), systemic sclerosis (SS), sjogren syndrome, rheumatoid arthritis (RA), Type 1 diabetes mellitus (T1D), Addison’s disease (AD), Behcet syndrome (BD), antiphospholipid syndrome, autoimmune hepatitis, vasculitis, polymyositis/dermatomyositis and myasthenia gravis. Cases were matched to controls in a 1:3 ratio by age and study period (the following 3 age-matched women with endometriosis and without history of autoimmune diseases).

For all included patients, we recorded age, medical present/past history, previous pelvic surgery, hormonal therapy at the time of inclusion in the study, smoking status, number of visits, time/type of surgery performed, histopathological diagnosis of endometriosis. Endometriotic lesions were classified according to their phenotype as ovarian endometrioma (OMA), deep infiltrating endometriosis (DIE) and superficial peritoneal endometriosis (SPE)^17^. Stage was assigned by medical surgery reports according to the revised American Society for Reproductive Medicine (ASRM)^18,19^ classification into minimal-mild endometriosis (stage 1–2) and moderate-severe endometriosis (stage 3–4). All participating patients gave an informed consent for their anonymized data to be used for research purposes (EndoGWA1, approved on 14th October 2014 by the Ethical Committee of the Ospedale San Raffaele in Milan, Istituto di Ricovero e Cura a Carattere Scientifico). Data collection followed the principles outlined in the Declaration of Helsinki.

### Statistical analysis

Data were analyzed using IBM SPSS Statistics (Version 24.0, Chicago, IL, USA). A p-value < 0.05 was considered to be statistically significant. A Shapiro–Wilk test was used to ascertain whether continuous variables had normal distribution. Assumption of homogeneity of variances was tested and satisfied based on Levene’s test when appropriate. Continuous and normally distributed variables were presented as mean ± standard deviation (SD), while continuous not normally distributed variables were expressed as median [25th–75th percentile] and categorical variables were presented as absolute values and percentages (%). The patients’ characteristics were compared between the group with a concomitant autoimmune disease (cases) and the group with a negative autoimmune status (controls) by use of a Person χ^2^ test or a Fisher’s exact test for qualitative variables and an independent Student’s t-test or a Mann–Whitney U test for quantitative variables as appropriate.

A logistic regression analysis was performed to determine the variables that could be independently associated with the presence of stage IV disease in endometriosis patients. Confounding factors were determined to be statistically significant at the threshold of p ≤ 0.05 by univariate analysis and were tested in a multiple logistic regression model. Odds ratios (OR) and their 95% confidence intervals (95% CI) were reported.

## Data Availability

The datasets generated during and/or analysed during the current study are available from the corresponding author on reasonable request.
