# Publisher Correction: Concomitant autoimmunity may be a predictor of more severe stages of endometriosis

**DOI:** 10.1038/s41598-021-97506-x

**Published:** 2021-08-31

**Authors:** Valeria Stella Vanni, Roberta Villanacci, Noemi Salmeri, Enrico Papaleo, Diana Delprato, Jessica Ottolina, Patrizia Rovere-Querini, Stefano Ferrari, Paola Viganò, Massimo Candiani

**Affiliations:** 1 Gynecology/Obstetrics Unit, IRCCS San Raffaele Scientific Institute, 20132 Milan, Italy; 2 Vita-Salute San Raffaele University, 20132 Milan, Italy; 3 Division of Immunology, Transplantation & Infectious Diseases, IRCCS San Raffaele Scientific Institute, 20132 Milan, Italy; 4 Reproductive Sciences Lab, Obstetrics and Gynecology Unit, IRCCS San Raffaele Scientific Institute, 20132 Milan, Italy

Correction to: *Scientific Reports* 10.1038/s41598-021-94877-z, published online 28 July 2021

The original version of this Article contained errors in the spelling of the authors Valeria Stella Vanni, Roberta Villanacci, Noemi Salmeri, Enrico Papaleo, Diana Delprato, Jessica Ottolina, Patrizia Rovere-Querini, Stefano Ferrari, Paola Viganò & Massimo Candiani which were incorrectly given as Vanni Valeria Stella, Villanacci Roberta, Salmeri Noemi, Papaleo Enrico, Delprato Diana, Ottolina Jessica, Rovere-Querini Patrizia, Ferrari Stefano, Viganò Paola & Candiani Massimo.

The original Article has been corrected.

